# Cyclovirobuxine D Induces Apoptosis and Mitochondrial Damage in Glioblastoma Cells Through ROS-Mediated Mitochondrial Translocation of Cofilin

**DOI:** 10.3389/fonc.2021.656184

**Published:** 2021-03-19

**Authors:** Lin Zhang, Ruoqiu Fu, Dongyu Duan, Ziwei Li, Bin Li, Yue Ming, Li Li, Rui Ni, Jianhong Chen

**Affiliations:** Department of Pharmacy, Daping Hospital, Army Medical University, Chongqing, China

**Keywords:** cyclovirobuxine D (CVBD), glioblastoma (GBM), apoptosis, mitochondrial damage, oxidative stress

## Abstract

**Background:**

Cyclovirobuxine D (CVBD), a steroidal alkaloid, has multiple pharmacological activities, including anti-cancer activity. However, the anti-cancer effect of CVBD on glioblastoma (GBM) has seldom been investigated. This study explores the activity of CVBD in inducing apoptosis of GBM cells, and examines the related mechanism in depth.

**Methods:**

GBM cell lines (T98G, U251) and normal human astrocytes (HA) were treated with CVBD. Cell viability was examined by CCK-8 assay, and cell proliferation was evaluated by cell colony formation counts. Apoptosis and mitochondrial superoxide were measured by flow cytometry. All protein expression levels were determined by Western blotting. JC-1 and CM-H_2_DCFDA probes were used to evaluate the mitochondrial membrane potential (MMP) change and intracellular ROS generation, respectively. The cell ultrastructure was observed by transmission electron microscope (TEM). Colocalization of cofilin and mitochondria were determined by immunofluorescence assay.

**Results:**

CVBD showed a greater anti-proliferation effect on the GBM cell lines, T98G and U251, than normal human astrocytes in dose- and time-dependent manners. CVBD induced apoptosis and mitochondrial damage in GBM cells. We found that CVBD led to mitochondrial translocation of cofilin. Knockdown of cofilin attenuated CVBD-induced apoptosis and mitochondrial damage. Additionally, the generation of ROS and mitochondrial superoxide was also induced by CVBD in a dose-dependent manner. N-acetyl-L-cysteine (NAC) and mitoquinone (MitoQ) pre-treatment reverted CVBD-induced apoptosis and mitochondrial damage. MitoQ pretreatment was able to block the mitochondrial translocation of cofilin caused by CVBD.

**Conclusions:**

Our data revealed that CVBD induced apoptosis and mitochondrial damage in GBM cells. The underlying mechanism is related to mitochondrial translocation of cofilin caused by mitochondrial oxidant stress.

## Introduction

Glioma, which is the most common central nervous system cancer, accounts for about 40% to 50% of all intracranial tumors (1). Glioblastoma (GBM), classified as grade IV by the World Health Organization (WHO), is the most fatal and malignant type of glioma (2). The median survival time of GBM patients is about 18 months, and only about 30% of patients achieve 2-year survival (3, 4). At present, the standard therapy for GBM is surgical resection combined with local radiotherapy, and adjuvant chemotherapy with the alkylating agent temozolomide (TMZ) (5). Although patients treated with TMZ have a significantly higher survival than those treated with radiotherapy alone, the overall prognosis is still poor, and the resistance of GBM cells to TMZ is often to blame (6, 7). Therefore, there is an urgent need to find a novel and effective anti-GBM agent.

Accumulated evidence has revealed that natural products have marvelous anti-cancer effects, such as inhibiting cell proliferation, inducing apoptosis and mitochondrial damage, and promoting oxidative-stress (8–12). Cyclovirobuxine D (CVBD) is an alkaloid derived from *Buxus sinica* and other plants of the same genus (13). Published studies suggest that CVBD has an effect on various cancer cells. For instance, CVBD inhibits colorectal cancer tumorigenesis *via* the CTHRC1-AKT/ERK-Snail signaling pathway (14), and exerts anticancer effects by suppressing the EGFR-FAK-AKT/ERK1/2-Slug signaling pathway in human hepatocellular carcinoma (15). Moreover, CVBD inhibits cell proliferation and induces mitochondria-mediated apoptosis in human gastric cancer cells (16), and induces autophagy-associated cell death *via* the Akt/mTOR pathway in MCF-7 human breast cancer cells (17). However, the anticancer effects and detailed mechanism of CVBD action against GBM have rarely been investigated.

Cofilin is from the actin-depolymerizing factor (ADF) family, which is best known as a key regulator of actin filament dynamics (18). Recent discoveries have increased our knowledge of cofilin beyond its well-characterized roles. Cofilin has a pivotal role in cancer progression, invasion, and apoptosis (19). Increasing evidence indicates that the function of cofilin is strongly associated with apoptosis mediated by mitochondrial functioning and dynamics (20). After induction of apoptosis, dephosphorylated cofilin is translocated from the cytosol into the mitochondria before the release of cytochrome c (21). The excessive generation of reactive oxygen species (ROS) can be harmful to cells, and cancer cells are more vulnerable to damage by increased oxidative stress caused by exogenous agents (22). ROS play a key role in cell growth, progression, differentiation, and death, especially the increased ROS involved in the fate of cancer cells (23, 24). Several studies have revealed that ROS participate in the activation of the classic apoptosis pathway and mitochondrial damage, through molecules and proteins associated with either mitochondrial function or cell death (25–28). Mitochondria are the main cellular organelles for bioenergetics, metabolism, biosynthesis, and cell death (29). Several reports have shown that the over-generation of mitochondrial superoxide could promote GBM cell death *via* phosphorylation of JNK (30). Mitochondrial superoxide accumulation could activate the intrinsic apoptosis pathway in multiple myeloma cells (31).

In our present study, the mechanism of CVBD for inducing mitochondrial damage and apoptosis in GBM cells was revealed. This work generates fresh insight into the association of CVBD-induced GBM cell apoptosis with the mitochondrial superoxide -mediated translocation of cofilin.

## Materials and Methods

### Chemicals and Antibodies

Cyclovirobuxine D (Cat.no. A0075) was purchased from CHENGDU MUST BIO-TECHNOLOGY CO., LTD (Chengdu, China); Z-VAD-FMK (Cat.no. HY-16658), Mitoquinone (Cat.no. HY-100116A) and CCCP (Cat.no. HY-100941) were purchased from MedChemExpress (Monmouth Junction, NJ, USA); N-acetyl-L-cysteine (NAC) (Cat.no. ST1546) was purchased from Beyotime (Shanghai, China); DMSO (Cat.no. D8418) was purchased from Sigma–Aldrich Chemical Co. (St. Louis, MO, USA); antibodies against C-Caspase3 (Cat.no. 9116S), GAPDH (Cat.no. 2118), phospho -AKT (Cat.no. 4060S) were purchased from Cell Signaling Technology (Beverly, MA, USA); cofilin (Cat.no. ab42824), phospho-Cofilin (S3, Cat.no. ab12866), VDAC1 (Cat.no. ab14734), phospho-ERK (Cat.no. ab50011), AKT (Cat.no. ab200195) and ERK (Cat.no. ab17942)were obtained from Abcam (Cambridge, UK); PARP (Cat.no. 13371-1-AP), and cytochrome c (Cat.no. 10993-1-AP) were purchased from Proteintech (Rosemont, IL, USA).

### Cell Lines and Cell Culture

The GBM cell lines, T98G and U251, were obtained from the American Type Culture Collection (ATCC, Manassas, VA, USA). Human astrocytes (HA) and growth medium were obtained from Scien Cell Research Laboratories (Carlsbad, CA, USA). DMEM medium (Cat.no. SH30022.01, Hyclone Laboratories, Inc. Logan, UT, USA) and 10% fetal bovine serum (Cat.no. 10099141C, Gibco, Carlsbad, CA, USA) were used for culturing the T98G and U251 cells. All cell lines were cultured in a humidified atmosphere at 37 °C in 5% CO_2_.

### CCK-8 Cell Viability Assay

Cells were seeded in 96-well plates with 5 × 10^3^ cells/well and incubated for 24 h, followed by treatment with CVBD (0, 40, 80, 120, 160 μM) for different periods of time (0, 12, 24, 36, 48 h). Additionally, the cells were treated with CVBD (120 μM) for 24 h after being pre-treated with NAC (20 μM) for 2 h or MitoQ (0.5 μM) for 30 min. Subsequently, a 10 μL Cell Counting Kit-8 (CCK-8, MCE, Cat.no. HY-K0301, Monmouth Junction, NJ, USA) was added to each well and incubated at 37 °C and 5% CO_2_ for 2 h before the absorbance at 450 nm was measured with a microplate reader (Agilent Technologies, CA, USA). The cell viabilities are expressed as a percentage.

### Cell Colony-Forming Assay

Cells were seeded in six-well plates with 500 cells/well and then incubated for 24 h, followed by treatment with different doses of CVBD (0, 40, 80, 120, 160 μM) for 24 h. The medium containing CVBD was then replaced with fresh complete medium and the cells were incubated at 37 °C and 5% CO_2_, changing the culture medium every three days. After 15 days, the colonies were fixed for 10 min with 4% paraformaldehyde (PFA) (Cat.no. C0121, Beyotime, Shanghai, China), followed by staining for 15 min with crystal violet staining solution (Cat.no. P0099, Beyotime, Shanghai, China). The colonies were scanned with CanoScan Lide (Canon, Japan).

### Apoptosis Assay

Cells were stained with Annexin V/FITC and PI (Cat.no. 556547, BD Biosciences, Franklin Lakes, NJ, USA) following the manufacturer’s specifications. Briefly, 2 × 10^5^ cells/well were collected and centrifuged at 600 g for 5 min. Cell pellets were resuspended with 1 mL PBS. Centrifugation and resuspension were repeated twice. Then, cells were stained with 2 μl Annexin V/FITC and 5 μl PI in 1× binding buffer for 15 min at room temperature in the dark. Quantitative analysis of apoptotic cells was undertaken by flow cytometry (Accuri C6, BD Biosciences, Franklin Lakes, NJ, USA).

### Measurement of Mitochondrial Membrane Potential (MMP)

Cells were seeded in six-well plates with 2 × 10^5^ cells/well and cultured for 24 h. Cells were stained with a JC-1 probe (Cat.no. C2006, Beyotime, Shanghai, China) according to the manufacturer’s instructions. After staining, cells were washed twice and 2 ml/well of medium was added. The green and red fluorescence were observed by fluorescence microscope (Thermo Fisher Scientific, Waltham, MA, USA). The level of mitochondrial membrane potential (MMP) is expressed as the relative ratio of red (J-aggregates) and green (monomer) fluorescence. Quantification of fluorescence intensity was obtained through analysis using Image J software (National Institutes of Health, Bethesda, MD, USA).

### Transmission Electronic Microscopy (TEM)

Cells were treated according to the previous method, and the collected cell pellets were fixed at 4 °C overnight in 2.5% glutaraldehyde, then were fixed at room temperature for 1.5 h in 2% osmium tetroxide using uranyl acetate/lead citrate to embed and stain the cells after fixation. The cell sections were carried out using a transmission electron microscope at 60 kV.

### RNA Interference Assay

The target shRNA sequence was constructed by Gene Chem Co. Ltd. (Shanghai, China). The sequence of the cofilin shRNA was as follows: 5′–CCGGAAGGTGTTCAATGACATGAAACTCGAGTTTCATGTCATTGAACACCTTTTTTTG–3′. The control shRNA plasmid was purchased from Santa Cruz Biotechnology, Inc. (Cat.no. sc-108060, Dallas, TX, USA). 293FT cells were co-transfected with the lentiviral packing vectors pLP1, pLP2, and pLP/VSVG (Invitrogen, Carlsbad, CA, USA), along with the shCofilin or shCon plasmid, using Lipofectamine 3000 (Invitrogen, Carlsbad, CA, USA) for 48 h. The supernatant containing the lentivirus was harvested and used for infection of T98G and U251 cells. Subsequently, selected stable cell lines were treated with 4 μg/ml puromycin (Cat.no. P9620. Sigma–Aldrich, St. Louis, MO, USA).

### Measurement of Intracellular ROS Generation

Cells were seeded in six-well plates with 2 × 10^5^ cells/well and incubated for 24 h. They were treated with the indicated concentrations of CVBD for 24 h, or pre-treated with NAC for 2 h before being treated with CVBD. Following replacement of the cell medium, we incubated the cells with 10 μM CM-H_2_DCFDA serum-free culture medium for 30 min at 37 °C (Cat.no. C6827, Molecular Probes, Thermo Fisher Scientific, Waltham, MA, USA). Subsequently, the cells were washed twice with PBS. The green fluorescence change of intracellular ROS was observed using a fluorescent microscope (Thermo Fisher Scientific, Waltham, MA, USA), and Image J software (National Institutes of Health, Bethesda, MD, USA) was used to quantify the ROS fluorescence intensity.

### Measurement of Mitochondrial Superoxide Generation

Cells were seeded in six-well plates with 2 × 10^5^ cells/well and cultured for 24 h. The cells were treated with the indicated concentrations of CVBD for 24 h, or were pre-treated with Mito Q for 30 min before being treated with CVBD. After the drug treatments, cells were incubated with MitoSOX™ Red (Invitrogen, Carlsbad, CA, USA) for 30 min at 37 °C. Quantification of mitochondrial superoxide was by flow cytometry (Accuri C6, BD Biosciences, Franklin Lakes, NJ, USA).

### Immunofluorescence

Cells were seeded on coverslips and cultured for 24 h. According to the manufacturer’s protocol, mitochondria were stained with MitoTracker (Deep Red FM) (Cat.no. M22426, Invitrogen, Carlsbad, CA, USA) for 30 min at 37 °C. Cells were fixed with 4% paraformaldehyde (PFA) for 30 min in the dark, and permeated with 0.1% Triton 100 (Cat.no. ST795, Beyotime, Shanghai, China) for 5 min. After being blocked in normal goat serum for 30 min at room temperature, cells were incubated with antibody overnight at 4 °C, followed by washing and incubating with the appropriate secondary antibodies. After immunostaining, cells were stained for 3–5 min with DAPI nuclear stain. Cells were observed using confocal microscopy (Cat.no. LSM 780NLO, Carl Zeiss, Germany).

### Extraction of Cell Mitochondria

Cells were seeded in 100mm cell culture dish and cultured for 24 h. Cell mitochondria isolation as performed using the Cell Mitochondria Isolation Kit (Cat.no. C3601, Beyotime, Shanghai, China) according to the manufacture’s protocol. Cells were collected and suspended with mitochondrial extraction agent. After being centrifugating, the precipitate was followed by washing twice with mitochondrial lysate agent. After being centrifugating again, the supernatant was collected as mitochondrial protein. Finally, the concentration of mitochondrial protein sample was quantified by BCA assay.

### Western Blotting

Cells were collected for lysis with RIPA buffer (Cat.no. P0013, Beyotime, Shanghai, China), and the concentration of each protein sample was quantified by BCA assay. The protein samples (15–30 μg) were separated by 10–12% SDS-PAGE and transferred to PVDF membranes (Cat.no. ISEQ00010, Millipore, Billerica, MA, USA). The membranes were incubated overnight with primary antibodies at 4 °C after being blocked with 5% milk for 1 h. Subsequently, the membranes were incubated with horseradish peroxidase (HRP)-conjugated antibodies for 2 h and visualized using an enhanced chemiluminescence (ECL) substrate (Cat.no. 1705060, Bio-Rad, Hercules, CA, USA). We used the Image J software (National Institutes of Health, Bethesda, MD, USA) to determine the gray density of the protein bands.

### Statistical Analysis

Colocalization correlation coefficients were determined using Image J software. All experimental data are represented as the mean ± SD from three independent experiments. The comparisons were performed using a one-way analysis of variance (ANOVA) or t-test by SPSS 19.0 (IBM Corporation, Armonk, NY, USA). * P < 0.05, ** P < 0.01 or *** P < 0.001 was considered statistically significant.

## Results

### CVBD Inhibits Cell Proliferation in Human GBM Cells

**Figure 1:**
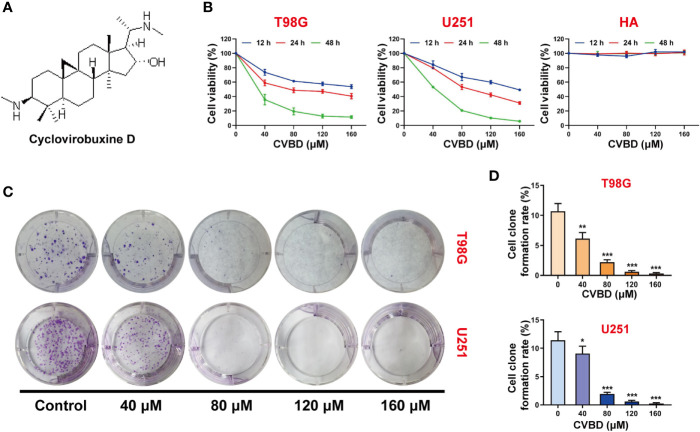
CVBD inhibits cell proliferation and colony formation in glioblastoma (GBM) cells. **(A)** The chemical structure of cyclovirobuxine D (CVBD). **(B)** GBM cells (T98G and U251) and normal human astrocytes (HA) were treated with CVBD (40, 80, 120, 160 μM) for 12, 24, and 48 h. Cell viability was measured by CCK-8 assay, and 0 μM CVBD was used as a control group. **(C, D)** Colony formation was assessed by plate clone formation assay in T98G and U251 cells (mean ± SD of three independent experiments, *P < 0.05, **P < 0.01, ***P < 0.001 compared with the control group).

The chemical structure of cyclovirobuxine D (CVBD) indicated in **Figure 1A**. To study the effect of CVBD on the growth of human GBM cells and normal human astrocytes (HA), the cell viabilities were determined by CCK-8 assay. We found that the cell viabilities were decreased in a dose-dependent manner in T98G and U251 cells treated with CVBD. The cell viability of normal human astrocytes (HA) was affected only rarely (**Figure 1B**). In addition, we also evaluated the effect of CVBD on cell clone formation in T98G and U251 cells. As shown in **Figures 1C, D**, treating T98G and U251 cells with CVBD clearly reduced cell clone formation in a dose-dependent manner. These results suggest that CVBD could inhibit GBM cell proliferation.

### CVBD Induces Apoptosis in Human GBM Cells

**Figure 2:**
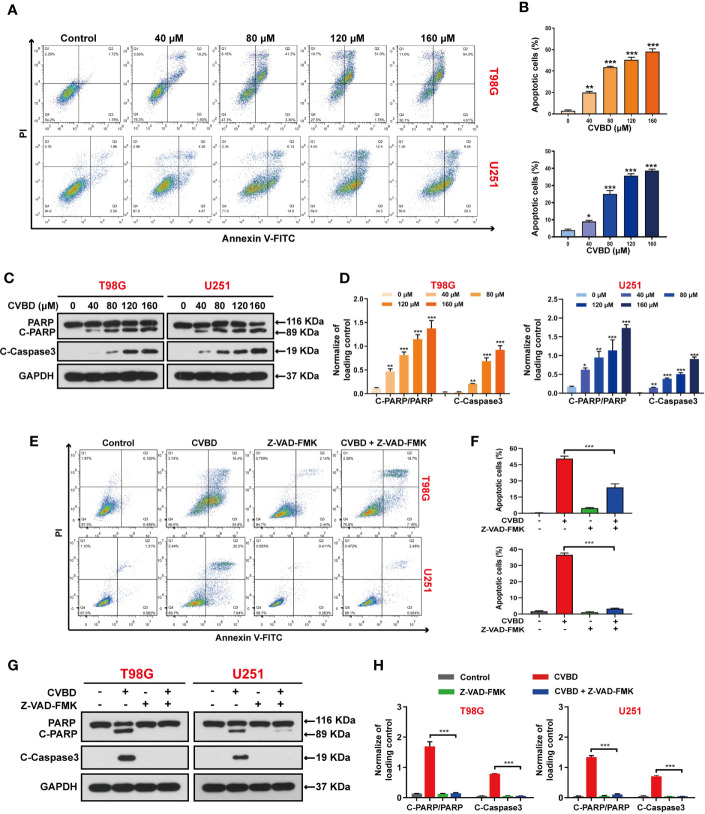
CVBD induces apoptosis in GBM cells. **(A, B)** T98G and U251 cells were exposed to various concentrations of CVBD (80, 120, 160 μM) for 24 h, and 0 μM CVBD was used as the control group. Apoptosis was detected by AnnexinV-FITC/PI staining and flow cytometry. **(C, D)** T98G and U251 cells were treated as indicated in **(A, B)**. The total cellular extracts of T98G and U251 cells were determined by Western blotting using antibodies against total PARP, cleaved PARP (C-PARP), and cleaved-Caspase3 (C-Caspase3) (mean ± SD of three independent experiments, *P < 0.05, **P < 0.01, ***P < 0.001 compared with the control group). GAPDH was used as a loading control. **(E, F)** T98G and U251 cells were pre-treated with Z-VAD-FMK (20 μM, 2 h) and post-treated with CVBD (120 μM) for 24 h. Apoptosis was detected by AnnexinV-FITC/PI staining and flow cytometry. **(G, H)** T98G and U251 cells were treated as indicated in **(E)**. The expression level of PARP, C-PARP, and C-Caspase3 were determined by Western blotting analysis. GAPDH was used as a loading control (mean ± SD of three independent experiments, ***P < 0.001 compared with CVBD treatment alone).

To determine whether CVBD causes GBM cell death, the apoptosis of T98G and U251 cells was detected by flow cytometry. Treatment of cells with CVBD for 24 h noticeably induced apoptosis of T98G and U251 cells in a dose-dependent manner (**Figures 2A, B**). Consistent with these findings, CVBD treatment caused activation of cleaved- caspase3 and degradation of PARP with formation of cleaved-PARP (**Figures 2C, D**). Furthermore, T98G and U251 cells were pre-treated with Z-VAD-FMK, a pan-caspase inhibitor, and this significantly attenuated the apoptotic rate induced by CVBD (**Figures 2E, F**). Western blotting results showed that pre-treatment with Z-VAD-FMK could markedly attenuate the upregulation of C-PARP and C-Caspase3 induced by CVBD, and inhibit the degradation of PARP induced by CVBD (**Figures 2G, H**). Overall, these results indicated that CVBD may induce caspase-dependent apoptosis of T98G and U251 cells.

### CVBD Induces Mitochondrial Damage in Human GBM Cells

**Figure 3:**
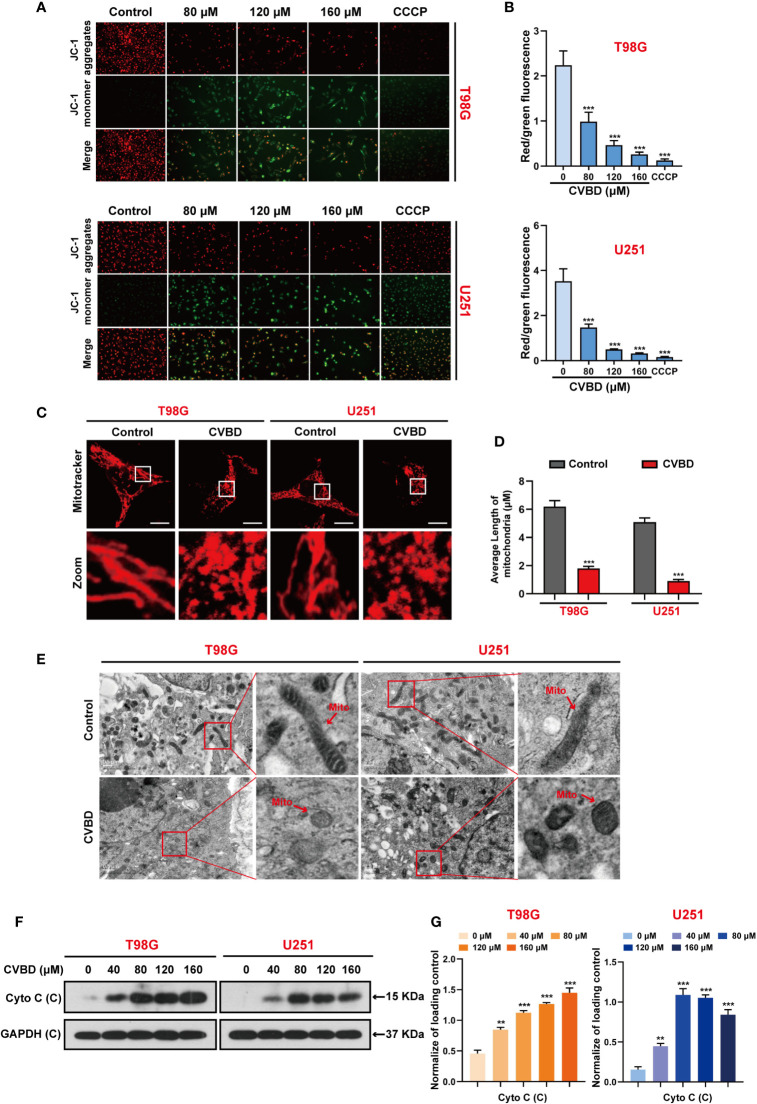
CVBD induces mitochondrial damage in GBM cells. **(A, B)** T98G and U251 cells were exposed to various concentrations of CVBD (80, 120, 160 μM) for 24 h, and 0 μM CVBD was used as the control group. CCCP was used as the positive control (50 μM, 30 min). The mitochondrial membrane potential (MMP) was detected by a fluorescence microscope using JC-1 probe staining (mean ± SD of three independent experiments ***P < 0.001 compared with the control group). **(C, D)** T98G and U251 cells were treated with CVBD (120 μM) for 24 h. Mitochondrial morphology was observed using MitoTracker (Deep Red FM) staining followed by confocal microscopy. Scale bars: 10 μM. The average length of the mitochondria was measured by Image J software (mean ± SD of three independent experiments, ***P < 0.001 compared with the control group). **(E)** Representative TEM images of T98G and U251 cells, treated as indicated in **(C)**. Red arrows indicate mitochondria. Scale bars: 0.5 μM. **(F, G)** T98G and U251 cells were treated with various concentrations of CVBD for 24 h. The expression level of Cyto C in the cytoplasm was detected by Western blotting analysis. GAPDH was used as a loading control (mean ± SD of three independent experiments, **P < 0.01, ***P < 0.001 compared with the control group).

Several studies have suggested that cell apoptosis can be caused by mitochondrial damage. To determine the effect of CVBD on mitochondrial damage in T98G and U251 cells, the mitochondrial membrane potential (MMP) was detected using a fluorescent probe JC-1.,and CCCP as an classical mitochondrial proton carrier uncoupling agent, which can increase the proton permeability of the mitochondrial membrane and destroy the mitochondrial membrane potential. As shown in **Figures 3A, B**, treatment with CVBD or CCCP enhanced the green fluorescence intensity and weakened the red fluorescence intensity in T98G and U251 cells, which indicates that the mitochondrial membrane potential (MMP) was decreased. Notably, the result of MitoTracker (Deep Red FM) staining showed the average length of mitochondria of cells exposed to CVBD was shorter than those exposed to placebo (control), which indicates that CVBD could lead to marked mitochondrial fragmentation (**Figures 3C, D**). To determine the morphology of mitochondria in T98G and U251 cells treated with CVBD, the ultrastructural changes of cells were examined by transmission electron microscopy (TEM). As shown in **Figure 3E**, the mitochondrial morphology of cells treated with CVBD was shrunken and rounded compared to the control group. The release of cytochrome c (Cyto C) from mitochondria into the cytoplasm indicated the activation of the intrinsic pathway of apoptosis, which mitochondrial dependent. Therefore, we evaluated the expression level of Cyto C in the cytosolic fraction by Western blotting analysis. As shown in **Figures 3F, G**, CVBD significantly increased the expression level of Cyto C in the cytosolic fraction. Together, these results show that CVBD induces mitochondrial damage in GBM cells.

### Mitochondrial Translocation of Cofilin Is Required for CVBD-Induced Mitochondrial Damage and Apoptosis in GBM Cells

**Figure 4:**
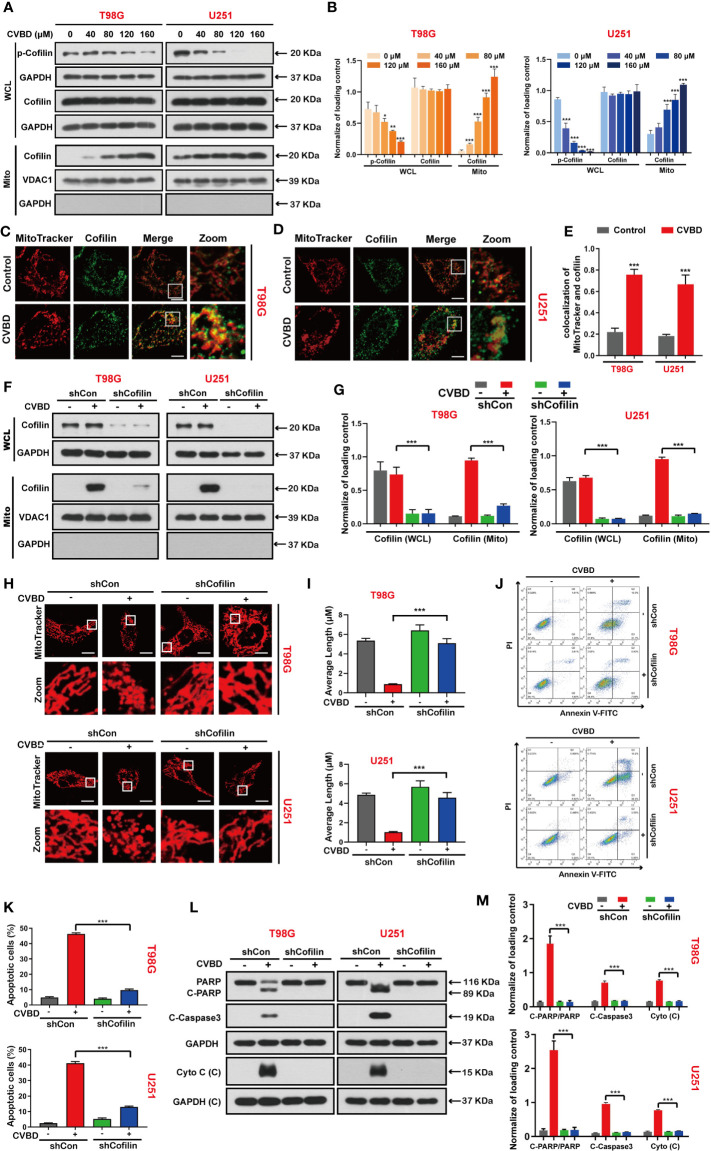
CVBD induces mitochondrial translocation of cofilin, and knockdown of cofilin attenuates CVBD-mediated mitochondrial damage and apoptosis. **(A, B)** T98G and U251 cells were exposed to various concentrations of CVBD for 24 h. The expression level of p-cofilin and cofilin in the whole cell lysate (WCL) and cofilin in the mitochondrial fractions was determined by Western blotting analysis. GAPDH and VDAC1 were used as loading controls. GAPDH was also used as cytosolic marker (mean ± SD of three independent experiments, *P < 0.05, **P < 0.01, ***P < 0.001 compared with the control group). **(C, D)** T98G and U251 were treated with CVBD (120 μM) for 24 h; the colocalization of MitoTracker (red) and cofilin (green) was observed by confocal microscopy. Scale bars: 10 μM. For **(E)** Quantitative analysis of colocalization of MitoTracker (red) and cofilin (green). Colocalization correlation coefficients were represented as mean ± SD (***P < 0.001 compared with the control group). For **(F–M)**, T98G and U251 cells were stably knocked down and exposed to CVBD (120 μM) for 24 h. **(F, G)** The expression level of cofilin in the WCL and mitochondrial fractions was determined by Western blotting. GAPDH and VDAC1 were used as loading controls. GAPDH was also used as cytosolic marker. **(H, I)** The mitochondrial morphology was observed using MitoTracker (Deep Red FM) staining, followed by confocal microscopy. Scale bars: 10 μM. The mitochondrial average length was measured with Image J software. **(J, K)** Apoptosis was detected by Annexin V-FITC/PI staining and flow cytometry. **(L, M)** The expression level of PARP, C-PARP, C-Caspase3, and Cyto C **(C)** was determined by Western blotting analysis. GAPDH was used as a loading control (mean ± SD of three independent experiments, ***P < 0.001).

We detected the effect of CVBD on de-phosphorylation and mitochondrial translocation of cofilin in T98G and U251 cells. Western blotting showed that CVBD decreased the expression level of phospho-cofilin in whole cell lysate (WCL), but increased the expression level of cofilin in the mitochondria fraction and decreased it in the cytosolic fraction in a dose-dependent manner (**Figures 4A, B**). To further confirm whether cofilin was translocated to mitochondria, the colocalization of cofilin and mitochondria was detected by immunofluorescence analysis. As shown in **Figures 4C, D**, the colocalization of the green puncta of cofilin and MitoTracker (Deep Red FM) was observed in cells treated with CVBD. The colocalization correlation coefficient of CVBD treatment group significantly higher than control group (**Figure 4E**).Overall, these results suggested that CVBD induced mitochondrial translocation of cofilin in GBM cells.

Based on the above studies, we further investigated whether mitochondrial translocation of cofilin is required for CVBD-induced mitochondrial damage and apoptosis. Therefore, we knocked down the cofilin with shRNA to clarify the role of cofilin in CVBD-induced mitochondrial damage and apoptosis. Western blotting results showed that knockdown of cofilin significantly inhibited the high expression level of cofilin in the mitochondria fraction induced by CVBD (**Figures 4F, G**). Knockdown of cofilin inhibited the mitochondrial fragmentation (**Figures 4H, I**) and attenuated the apoptosis induced by CVBD (**Figures 4J, K**). Consistent with these findings, knockdown of cofilin reduced the CVBD-mediated upregulation of C-PARP and C-Caspase3 and the release of Cyto C from the mitochondria, and inhibited the degradation of PARP induced by CVBD (**Figures 4L, M**). Taken together, these results suggest that mitochondrial translocation of cofilin is essential for CVBD-induced mitochondrial damage and apoptosis.

### CVBD Induces Accumulation of Reactive Oxygen Species (ROS) in GBM Cells

**Figure 5:**
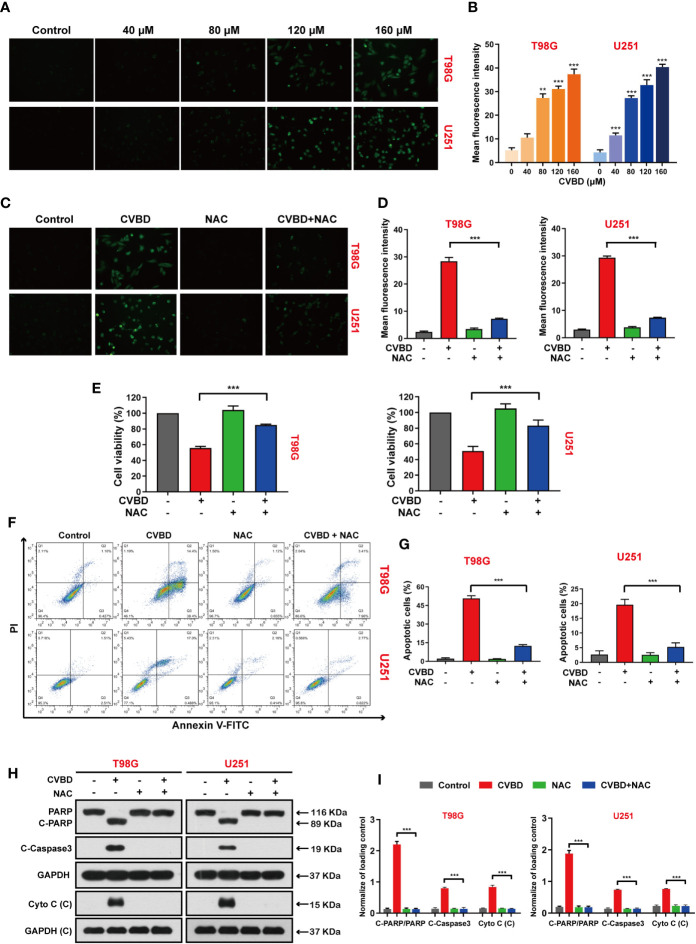
CVBD induces ROS generation and NAC inhibits ROS generation, attenuating CVBD-mediated apoptosis. **(A, B)** T98G and U251 cells were exposed to various concentrations of CVBD for 24 h, and 0 μM CVBD was used as the control group. The ROS generation was observed by fluorescence microscopy using a CM-H_2_DCFDA probe (mean ± SD of three independent experiments, **P < 0.01, ***P < 0.001 compared with the control group). **(C, D)** T98G and U251 cells were pre-treated with NAC (20 μM, 2 h) and post-treated with CVBD (120 μM) for 24 h, and the ROS generation was observed by fluorescence microscopy using CM-H_2_DCFDA probe staining. **(E)** T98G and U251 cells were treated as indicated in **(C)**, and the cell viability was examined by CCK-8 assay. **(F, G)** T98G and U251 cells were treated as indicated in **(C)**, and apoptosis was detected by AnnexinV-FITC/PI staining and flow cytometry. **(H, I)** T98G and U251 cells were treated as indicated in **(C)**, and the expression of PARP, C-PARP, C-Caspase3, and Cyto C **(C)** was determined by Western blotting analysis. GAPDH was used as a loading control (mean ± SD of three independent experiments, ***P < 0.001 compared with CVBD treatment alone).

We explored the effect of CVBD on ROS generation in GBM cells, in which the green fluorescence of the CM-H_2_DCFDA probe indicated intracellular ROS generation. As shown in **Figures 5A, B**, green fluorescence was enhanced in a dose-dependent manner in cells treated with CVBD. Subsequently, cells pre-treated with N-acetyl-L-cysteine (NAC) showed lower green fluorescence intensity than those treated with CVBD alone (**Figures 5C, D**). In **Figure 5E**, the cell viability of cells pre-treated with NAC is higher than in those treated with CVBD alone. To further investigate whether ROS were involved in CVBD-induced cell apoptosis, cells were pre-treated with NAC for 2 h, and apoptosis was detected by flow cytometry. As shown in **Figures 5F, G**, cells pre-treated with NAC significantly decreased the apoptotic rate induced by CVBD. Western blotting results showed that pre-treatment with NAC significantly obstructed the CVBD-induced upregulation of C-PARP and C-Caspase3 and the release of Cyto C, and inhibited the degradation of PARP induced by CVBD (**Figures 5H, I**). These findings indicated that intracellular ROS accumulation resulted in CVBD-meditated apoptosis in GBM cells.

### CVBD Induces Mitochondrial Superoxide Production in GBM Cells

**Figure 6:**
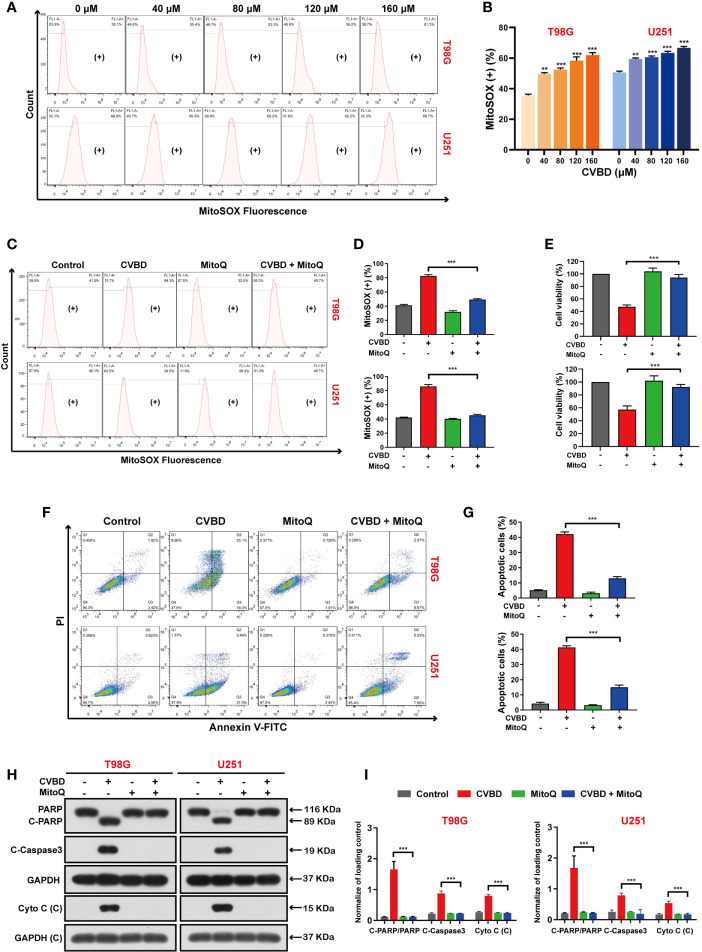
CVBD induces mitochondrial superoxide generation and MitoQ inhibits mitochondrial superoxide production, which attenuates CVBD-mediated apoptosis. **(A, B)** T98G and U251 cells were exposed to various concentrations of CVBD for 24 h, and 0 μM CVBD was used as the control group. The mitochondrial superoxide generation was detected by flow cytometry using MitoSOX™ Red staining (mean ± SD of three independent experiments, **P < 0.01, ***P < 0.001 compared with the control group). **(C, D)** T98G and U251 cells were pre-treated with MitoQ (0.5 μM, 30 min) and post-treated with CVBD (120 μM) for 24 h. The mitochondrial superoxide generation was detected by flow cytometry using MitoSOX™ red staining. **(E)** T98G and U251 cells were treated as indicated in **(C)**, and the cell viability was examined by CCK-8 assay. **(F, G)** T98G and U251 cells were treated as indicated in **(C)**, and the apoptosis was detected by AnnexinV-FITC/PI staining and flow cytometry. **(H, I)** T98G and U251 cells were treated as indicated in **(C)**, and the expression of PARP, C-PARP, C-Caspase3, and Cyto C **(C)** was determined by Western blotting analysis. GAPDH was used as a loading control (mean ± SD of three independent experiments, **P < 0.01, ***P < 0.001 compared with CVBD treatment alone).

To further clarify the role of mitochondrial oxidative stress in apoptosis induced by CVBD, mitochondrial superoxide production was detected by MitoSOX™ Red staining and flow cytometry. As shown in **Figures 6A, B**, the percentage of mitochondrial superoxide production in GBM cells was gradually increased after CVBD treatment. Subsequently, cells pre-treated with MitoQ showed a lower percentage of mitochondrial superoxide than those treated with CVBD alone (**Figures 6C, D**). In **Figure 6E**, pretreatment with MitoQ, a mitochondrial superoxide inhibitor, reversed the decrease in cell viability induced by CVBD. To further investigate whether Mito ROS are involved in CVBD-induced cells apoptosis, cells were pre-treated with MitoQ and apoptosis was detected by flow cytometry. As shown in **Figures 6F, G**, pre-treatment with MitoQ significantly decreased the apoptotic rate induced by CVBD. Consistent with the above findings, Western blotting results showed that pre-treatment with MitoQ significantly reduced the upregulation of C-PARP and C-Caspase3 and the release of Cyto C induced by CVBD, and could inhibit the degradation of PARP induced by CVBD (**Figures 6H, I**). These results suggested that apoptosis of T98G and U251 induced by CVBD was caused by mitochondrial superoxide production.

### Inhibited Mitochondrial Superoxide Production Blocked CVBD-Meditated Mitochondrial Translocation of Cofilin

**Figure 7:**
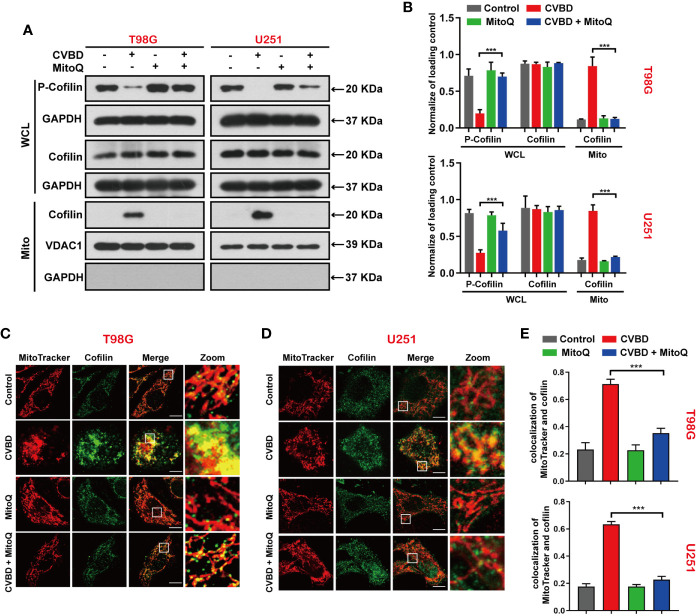
Inhibited Mitochondrial superoxide production attenuates CVBD-induced mitochondrial translocation of cofilin. **(A, B)** T98G and U251 cells were pre-treated with MitoQ (0.5 μM, 30 min) and post-treated with CVBD (120 μM) for 24 h. The expression level of p-cofilin and cofilin in the whole cell lysate (WCL), and cofilin in the mitochondrial fractions, was determined by Western blotting analysis. GAPDH and VDAC1 were used as loading controls. GAPDH was also used as cytosolic marker (mean ± SD of three independent experiments, ***P < 0.001 compared with CVBD treatment alone). **(C, D)** T98G and U251 cells were treated as indicated in **(A)**, and the colocalization of MitoTracker (red) and cofilin (green) was observed by confocal microscopy. Scale bars: 10 μM. **(E)** Quantitative analysis of colocalization of MitoTracker (red) and cofilin (green). Colocalization correlation coefficients were represented as mean ± SD (***P < 0.001 compared with CVBD treatment alone).

To investigate whether the generation of mitochondrial superoxide is related to the mitochondrial translocation of cofilin induced by CVBD, we used Western blotting and an immunofluorescence assay to evaluate the mitochondrial translocation of cofilin. In **Figures 7A, B**, Western blotting results show that pre-treatment with MitoQ markedly decreased the CVBD-induced upregulation of cofilin in the mitochondria fraction, and reversed the downregulation of p-cofilin in the whole cell lysate (WCL). We subsequently determined the colocalization of cofilin and mitochondria by immunofluorescence assay. As shown in **Figures 7C, D**, pre-treatment with MitoQ obviously decreased the CVBD-mediated colocalization of cofilin’s green puncta and MitoTracker (Deep Red FM). The colocalization correlation coefficient of pre-treatment with MitoQ group significantly lower than CVBD treatment alone (**Figure 7E**). These results indicated that inhibited mitochondrial superoxide generation blocked CVBD-meditated mitochondrial translocation of cofilin.

## Discussion

Cyclovirobuxine D (CVBD), a steroidal alkaloid extracted from *Buxus sinica*, has a long history of treating cardiovascular diseases (13). However, studies are increasingly reporting that CVBD shows remarkable suppressive effects in various cancers, such as colorectal cancer, hepatocellular carcinoma, gastric cancers, and breast cancer. In terms of mechanism, several studies have found that CVBD, through the CTHRC1-AKT/ERK-Snail pathway or EGFR-FAK-AKT/ERK1/2-Slug pathway, inhibit cancer tumorigenesis through CVBD inducing mitochondrial apoptosis in cancer cells. Moreover, CVBD causes cancer cell death of autophagy associated with the AKT/mTOR pathway (14–17). Meanwhile, CVBD could active AKT/ERK signaling pathway in T98G and U251. In our current study, we found that CVBD inhibited proliferation and induced apoptosis of GBM cells. Mechanistically, we further found that CVBD triggered apoptosis by causing mitochondrial dysfunction, which is primarily attributed to the mitochondria translocation of cofilin *via* the activation of mitochondrial oxidative stress.

As previous published studies have mentioned, cofilin is best known as an actin depolymerizing factor, and it could increase the rate of actin depolymerization through regulating actin dynamics (32, 33). Several studies reveal that cofilin, a member of the cofilin/actin depolymerizing factor (ADF) family, plays a critical role in mitochondrial function and apoptosis. Recently, it was reported that de-phosphorylation and mitochondrial translocation of cofilin are connected with mitochondrial damage (34). Cofilin is also involved in the malignant invasion of cancer cells (35), and has been identified as a key protein involved in the initiation phase of oxidative stress-mediated mitochondrial apoptosis (21, 36). Recent research suggested that mitochondrial translocation of cofilin is an early stage in cell apoptosis (21). Mitochondrial translocation of cofilin induced by cyclohexene was found to be the underlying mechanism of anti-leukemia (37). Cofilin was confirmed to translocate to the mitochondria after isoalantolactone-induced apoptosis in GBM (38). These results indicated that mitochondrial translocation of cofilin is pivotal for inducing cell apoptosis. However, only dephosphorylated cofilin could translocate to the mitochondria and lead to mitochondrial damage and cell apoptosis (21). During apoptosis, the dephosphorylated expression level of cofilin is increased, and cofilin is translocated from the cytosol to the mitochondria, resulting in cytochrome c release and activating caspase-dependent apoptosis (39). Consistent with these reports, our study confirmed that cofilin dephosphorylates and translocates to the mitochondria during CVBD-induced apoptosis, leading to mitochondrial damage. CVBD treatment decreased the expression level of phospho-cofilin in the whole cell lysate (WCL) and increased the expression level of cofilin in the mitochondria fraction. Knocking down cofilin reduced the mitochondrial translocation of cofilin induced by CVBD. Third, knocking down cofilin weakened the apoptosis induced by CVBD, reduced the upregulation of cleaved-PARP and cleaved-Caspase3 expression levels, and inhibited the release of cytometry c from the mitochondria mediated by CVBD. Therefore, these results further support the idea that mitochondrial translocation of cofilin is required for CVBD-induced apoptosis.

It has been demonstrated that ROS play a key role in cell proliferation and differentiation (40, 41), but excessive generation of ROS can lead to oxidative damage of lipids, proteins, and DNA (42). ROS and oxidant stress of cells have been associated with cancer; increased ROS plays an important part in initiation and progression of cancers (23, 43). Previous reports have suggested that excessive production of ROS by exogenous means is more harmful to cancer cells (22). For instance, increasing ROS by exogenous drugs can cause apoptosis, DNA damage, or mitochondrial dysfunction of several cancers, including colorectal cancer, oral cancers, and breast cancer cells (44–46). We found that the effect of CVBD in inducing GBM cell apoptosis is related to the increase of intracellular ROS. First, CVBD increased the level of intracellular ROS in a dose-dependent manner. Second, NAC, as a ROS inhibitor, exhibited clear effects by decreasing the level of intracellular ROS induced by CVBD. Third, NAC pre-treatment significantly attenuated the CVBD-induced apoptosis in T98G and U251 cells. Thus, we explicated that CVBD-induced apoptosis of GBM occurs by upregulating the level of intracellular ROS.

Mitochondria play a pivotal role in cellular energy metabolism and cell apoptosis (29, 47). In the literature we found that approximately 90% of intracellular ROS is produced by the mitochondria, which are the main source of superoxide radicals (48, 49). Excessive mitochondrial oxidant stress could lead to cytochrome c release from the mitochondria to the cytosol, and activate caspase-apoptosis and DNA damage in cancer cells (50). Mitochondrial superoxide production results in caspase activation and pancreatic cancer cell apoptosis (51, 52). Scavenging MitoSOX could effectively attenuate the mitochondria impairment and apoptosis induced by DOX (53, 54). Consistent with these findings, in our study we found that the effect of CVBD in inducing GBM cell apoptosis is related to the increase of mitochondrial superoxide. First, the level of mitochondrial superoxide was increased by CVBD treatment in a concentration-dependent manner. Second, MitoQ, as a mitochondrial–targeted antioxidant, inhibited the generation of mitochondrial superoxide aroused by CVBD. Third, pre-treatment with MitoQ also significantly weakened the apoptosis caused by CVBD in T98G and U251 cells. These results could be explained by the fact that inhibition of the production of mitochondrial superoxide can largely suppress the apoptosis-inducing effect of CVBD. We also clarified the relationship between the generation of ROS and mitochondrial superoxide with apoptosis induced by CVBD. In light of this, we speculated whether ROS or mitochondrial superoxide production can give rise to the mitochondrial translocation of cofilin. A recent study demonstrated that methyl antcinate A (MAA) activated the generation of ROS in Huh7 cells, and that MAA led to mitochondrial translocation of cofilin. Pre-treatment of Huh7 cells with NAC markedly attenuated the apoptosis and mitochondrial translocation of cofilin induced by MAA (55). However, whether mitochondrial superoxide production is the reason for mitochondrial translocation of cofilin has not yet been studied. Surprisingly, we found that the level of cofilin in the mitochondria in GBM cells was decreased following pre-treatment with MitoQ, compared to CVBD treatment alone. Likewise, pre-treatment with MitoQ led to the colocalization of cofilin and mitochondria being dramatically inhibited. These results indicated that mitochondrial superoxide production could lead to the mitochondrial translocation of cofilin.

## Data Availability Statement

The datasets presented in this study can be found in online repositories. The names of the repository/repositories and accession number(s) can be found in the article/**Supplementary Material**.

## Author Contributions

Conceptualization, RF, BL and JC. Methodology, RF, LZ, BL, DD, LL and ZL. Experiments and data curation, LZ, RF, DD, YM, RN, and ZL. Writing—original draft preparation, LZ and RF. Writing—review and editing, RF, BL and JC. Supervision, ZL and JC. All authors contributed to the article and approved the submitted version.

## Funding

This research was funded by the project of Science and Health Combined Traditional Chinese Medicine of Chongqing (grant no. ZY201801004).

## Conflict of Interest

The authors declare that the research was conducted in the absence of any commercial or financial relationships that could be construed as a potential conflict of interest.
