# Supplementary figures and images for: Cyclovirobuxine D Induces Apoptosis and Mitochondrial Damage in Glioblastoma Cells Through ROS-Mediated Mitochondrial Translocation of Cofilin
Source: Front Oncol. 2021 Mar 19;11:656184. doi: 10.3389/fonc.2021.656184 (PMC8018288; doi:10.3389/fonc.2021.656184)

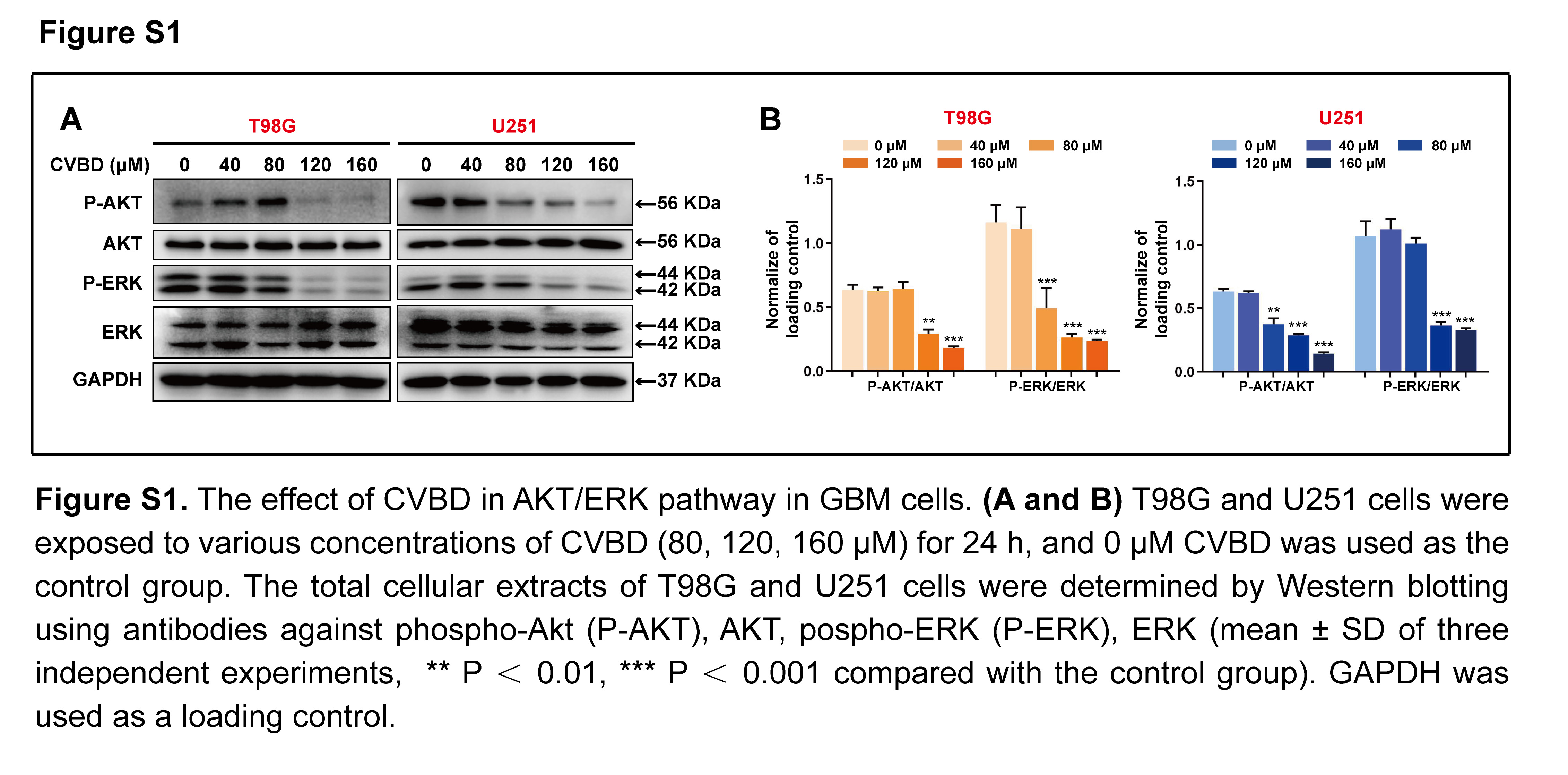

Supplement: Supplementary file 1 [file Image_1.jpg]
